# Exploring the intersection: Peptic ulcers and hemolysis—Unraveling the complex relationship

**DOI:** 10.1097/MD.0000000000037565

**Published:** 2024-03-15

**Authors:** Emmanuel Ifeanyi Obeagu, Getrude Uzoma Obeagu

**Affiliations:** a Department of Medical Laboratory Science, Kampala International University, Kampala, Uganda; b School of Nursing Science, Kampala International University, Kampala, Uganda.

**Keywords:** anemia, hemolysis, peptic ulcers, red blood cell

## Abstract

This paper investigates the intriguing relationship between peptic ulcers and hemolysis, 2 seemingly distinct medical conditions, aiming to unravel their potential interconnections and clinical implications. While traditionally studied in isolation, recent evidence has surfaced suggesting possible links and shared mechanisms between these conditions. This paper explores the underlying pathophysiological associations, shared risk factors, diagnostic challenges, management strategies, and implications for clinical practice and health policy. The interplay between peptic ulcers and hemolysis stems from shared inflammatory pathways, notably attributed to *Helicobacter pylori* infection in peptic ulcers, which might trigger systemic inflammatory responses contributing to hemolysis. Common risk factors including genetic predispositions, autoimmune disorders, and medication use (such as nonsteroidal anti-inflammatory drugs) are implicated in the development of both peptic ulcers and hemolytic conditions, suggesting a potential convergence of these disorders in affected individuals. Diagnostic considerations pose challenges, as overlapping symptoms and laboratory findings may complicate accurate differentiation between peptic ulcers and hemolysis. Recognizing the potential interplay between peptic ulcers and hemolysis holds significant implications for clinical practice and health policy. Streamlining diagnostic algorithms, fostering interdisciplinary collaborations, and developing tailored guidelines are pivotal in optimizing patient care. Continued research efforts, collaborative clinical approaches, and informed health policies are essential in advancing our understanding and enhancing patient care for individuals navigating the intersection of peptic ulcers and hemolysis.

## 1. Introduction

Peptic ulcers and hemolysis represent distinct yet clinically significant conditions that have traditionally been explored within the domains of gastroenterology and hematology, respectively.^[1]^ Peptic ulcers, characterized by mucosal erosions in the gastrointestinal tract, and hemolysis, involving the premature destruction of red blood cells (RBCs), have garnered considerable attention in medical research and clinical practice. However, recent insights suggest potential interconnections between these seemingly disparate entities, prompting a reexamination of their relationship and shared implications.^[2]^ Peptic ulcers, primarily associated with *Helicobacter pylori (H pylori*) infection and nonsteroidal anti-inflammatory drug (NSAID) use, have been extensively studied for their impact on gastrointestinal health.^[3]^ Conversely, hemolysis, encompassing a spectrum of conditions from inherited disorders to acquired diseases, has long been a focus of hematological investigation. Recent evidence indicates possible links between peptic ulcers and hemolysis, revealing intriguing associations in pathophysiological mechanisms and shared risk factors. Chronic inflammation, notably induced by *H pylori* infection in peptic ulcers, has been implicated in triggering systemic inflammatory responses that might extend beyond the gastrointestinal tract, potentially affecting RBC integrity and contributing to hemolysis.^[4]^

Furthermore, common predisposing factors, including genetic susceptibilities, autoimmune conditions, and medication usage, have emerged as shared elements in the development and progression of both peptic ulcers and hemolytic disorders. These shared risk factors suggest a potential convergence of these conditions in affected individuals, prompting a reconsideration of their conventional categorizations. Diagnostic challenges arise in accurately differentiating between peptic ulcers and hemolytic disorders due to overlapping clinical presentations and laboratory findings, necessitating comprehensive evaluations for precise identification and tailored management.^[5]^ This paper endeavors to delve into the complex relationship between peptic ulcers and hemolysis, exploring potential pathophysiological links, shared risk factors, diagnostic intricacies, management strategies, and implications for clinical practice and health policy. Understanding the intersection of these conditions holds promise for advancing knowledge, refining diagnostic approaches, and optimizing therapeutic interventions for individuals navigating the confluence of peptic ulcers and hemolysis.

## 2. Peptic ulcer

A peptic ulcer refers to a sore or lesion that develops on the lining of the stomach, the lower part of the esophagus, or the upper portion of the small intestine, known as the duodenum. These ulcers occur when the protective lining of these areas is eroded, exposing the underlying tissues to stomach acid and digestive enzymes.^[6]^ The primary causes of peptic ulcers include infection with *H pylori* bacteria, long-term use of NSAIDs like ibuprofen or aspirin, excessive stomach acid production, and lifestyle factors such as smoking and excessive alcohol consumption.^[7]^ Common symptoms of peptic ulcers include abdominal pain, often described as burning or gnawing and typically occurring between meals or during the night. Other symptoms may include nausea, vomiting, bloating, weight loss, and appetite changes. Diagnosis involves various methods such as upper endoscopy (gastroscopy), where a thin tube with a camera is inserted into the stomach to visualize ulcers, and tests to detect *H pylori* infection, including blood, stool, or breath tests.^[8]^ Treatment strategies aim to alleviate symptoms, heal the ulcer, and prevent recurrence. This can involve antibiotics to eradicate *H pylori*, medications to reduce stomach acid production (proton pump inhibitors and histamine H2-receptor antagonists), and lifestyle changes like quitting smoking and avoiding NSAIDs.^[9]^ If left untreated, peptic ulcers can lead to complications such as bleeding, perforation (where the ulcer creates a hole through the stomach or duodenal wall), and gastric outlet obstruction (when inflammation or scarring blocks the passage of food). Preventive measures include avoiding NSAIDs, managing stress, limiting alcohol consumption, avoiding smoking, and adhering to prescribed medications to control stomach acid production.^[10]^ Peptic ulcers are common and can often be effectively treated with medications and lifestyle modifications. Timely diagnosis and appropriate management are crucial in preventing complications and promoting healing.^[11]^

## 3. Hemolysis

Hemolysis refers to the premature destruction or breakdown of RBCs either within the bloodstream or within organs, leading to the release of hemoglobin. This process can occur due to various factors and conditions, resulting in the impairment of RBCs’ normal lifespan and function.^[12]^ Hemolysis can be caused by a wide range of factors, including inherited disorders such as sickle cell anemia, thalassemia, and hereditary spherocytosis. Acquired conditions such as autoimmune disorders (like autoimmune hemolytic anemia), infections, certain medications, blood transfusion reactions, toxins, mechanical damage (e.g., heart valve defects), and metabolic disorders can also trigger hemolysis.^[13]^ The symptoms of hemolysis can vary depending on the underlying cause and the rate of RBC destruction. Common symptoms include fatigue, weakness, pallor (pale skin), jaundice (yellowing of the skin and eyes), dark urine (due to increased bilirubin), and in severe cases, symptoms of anemia such as shortness of breath and rapid heart rate.^[14]^ Diagnosing hemolysis involves a combination of clinical assessment, laboratory tests, and sometimes specialized studies. Tests may include a complete blood count (CBC), reticulocyte count (to assess RBC production), peripheral blood smear, bilirubin levels, haptoglobin levels, and direct and indirect Coombs tests to identify autoimmune causes.^[15,16]^ Treatment of hemolysis depends on the underlying cause. In some cases, addressing the underlying condition or discontinuing medications causing hemolysis may be sufficient. In other instances, treatment may involve medications to suppress the immune system (in autoimmune hemolytic anemia), blood transfusions, or, in severe cases, removal of the spleen (splenectomy). Severe or chronic hemolysis can lead to complications such as anemia, increased risk of gallstones (due to excess bilirubin), and potential organ damage in cases of significant RBC destruction.^[17]^ Preventing hemolysis involves managing underlying conditions effectively, avoiding triggers known to cause hemolysis, and in certain cases, taking precautions during blood transfusions to prevent transfusion reactions.^[18]^ Hemolysis can be a manifestation of various diseases and conditions, necessitating a thorough evaluation by healthcare professionals to identify the underlying cause and provide appropriate treatment. Prompt diagnosis and management are crucial to prevent complications and improve outcomes for individuals affected by hemolysis.

## 4. Pathophysiological links

The pathophysiological links between peptic ulcers and hemolysis, though seemingly disparate, involve intricate interconnections that have drawn attention due to potential shared mechanisms and systemic effects. While primarily affecting distinct anatomical systems—the gastrointestinal tract in peptic ulcers and the hematological system in hemolysis—recent insights suggest potential overlapping pathways and interactions.^[19]^ Peptic ulcers, often associated with *H pylori* infection or NSAID use, induce local and systemic inflammation. Chronic inflammation, triggered by these factors, could contribute to a systemic inflammatory state, potentially impacting RBCs. Inflammatory mediators and oxidative stress released in response to peptic ulcers might affect RBC integrity and survival, thereby influencing hemolytic processes.^[20]^ The pro-inflammatory milieu associated with peptic ulcers, characterized by increased production of reactive oxygen species and inflammatory cytokines, may have implications for RBC homeostasis. Oxidative stress can cause RBC membrane damage, leading to premature RBC destruction or susceptibility to hemolysis. Additionally, the generation of free radicals might affect RBC lifespan and function.^[21]^ Chronic inflammation and RBC damage resulting from peptic ulcers might contribute to alterations in hemoglobin breakdown and bilirubin metabolism. Increased hemolysis, if influenced by the inflammatory cascade initiated by peptic ulcers, can elevate circulating levels of bilirubin. Elevated bilirubin concentrations could impact liver function and, in severe cases, contribute to jaundice.^[22]^ Immunological aberrations associated with peptic ulcers, especially in cases of autoimmune gastritis, might contribute to autoimmune phenomena affecting RBCs. Autoimmune-mediated mechanisms, though primarily linked to the gastric mucosa, could extend to RBCs, potentially resulting in autoimmune hemolytic anemia or related conditions. Peptic ulcers, particularly in cases of bleeding ulcers or severe complications, might result in microvascular alterations. Changes in microcirculation and endothelial dysfunction, triggered by ulcer-related complications or inflammatory responses, might impact RBC integrity or exacerbate conditions predisposing individuals to hemolysis.^[23]^ Understanding these potential pathophysiological links between peptic ulcers and hemolysis provides a conceptual framework for exploring shared mechanisms and systemic effects. While current evidence is preliminary, further research is warranted to elucidate the precise connections and clinical implications, potentially offering new insights into the management and holistic approach to these seemingly distinct medical conditions.

## 5. Shared risk factors

Peptic ulcers and hemolysis, although conventionally considered distinct medical conditions, may share certain risk factors that contribute to their development or exacerbation. Exploring these shared risk factors offers insights into potential overlaps and commonalities between these seemingly unrelated conditions.^[24]^ Certain medications are known to predispose individuals to both peptic ulcers and hemolysis. NSAIDs, including aspirin and ibuprofen, commonly used for pain relief or as antiplatelet agents, can cause gastrointestinal irritation, leading to peptic ulcers. Simultaneously, NSAIDs have been associated with drug-induced immune hemolytic anemia, particularly in susceptible individuals.^[25]^ *H pylori* infection is a well-established risk factor for peptic ulcers. This bacterial infection can trigger gastric inflammation and ulcer formation. Recent studies have suggested potential associations between *H pylori* infection and alterations in immune responses, which might contribute to hemolytic conditions, although the precise mechanisms remain under investigation.^[26,27]^ Certain autoimmune conditions, such as systemic lupus erythematosus and rheumatoid arthritis, are linked to an increased risk of both peptic ulcers and hemolytic disorders. Autoimmune phenomena associated with these conditions can predispose individuals to gastrointestinal mucosal damage, leading to ulcers, while also affecting RBCs, potentially causing autoimmune hemolytic anemia or other hemolytic conditions.^[28]^ Inherited conditions affecting RBC structure or function, such as hereditary spherocytosis, thalassemia, or sickle cell disease, predispose individuals to hemolysis. While these conditions primarily impact RBCs, there is evidence suggesting an association between genetic factors influencing inflammatory responses and susceptibility to gastrointestinal disorders, including peptic ulcers.^[29]^ Chronic inflammatory states, whether originating from autoimmune disorders, chronic infections, or persistent exposure to environmental factors, might serve as a common underlying factor predisposing individuals to both peptic ulcers and hemolysis. Inflammatory responses, when sustained, can affect multiple physiological systems, potentially impacting gastrointestinal integrity and RBC homeostasis. Lifestyle factors, including stress, smoking, excessive alcohol consumption, and poor dietary habits, have been implicated in peptic ulcer development. While their direct association with hemolysis may be less clear, these factors can exacerbate physiological stressors and immune responses, potentially influencing RBC integrity indirectly. Understanding these shared risk factors provides a basis for recognizing potential intersections between peptic ulcers and hemolysis. While each condition manifests differently, individuals with overlapping risk profiles may be at higher susceptibility for both, necessitating comprehensive evaluations and tailored management strategies to address these combined risks effectively. Table 1 shows for risk factors and management strategies.

**Table 1:** for risk factors and management strategies.

Risk factors	Management strategies
Peptic ulcers	
*H pylori* infection	—Antibiotic therapy to eradicate *H pylori* —Proton pump inhibitors (PPIs) for acid suppression
NSAID use	—Limit or avoid NSAIDs, consider alternative pain management —PPIs for gastroprotection in individuals requiring NSAIDs
Excessive stomach acid production	—PPIs or H2 blockers to reduce gastric acid secretion —Lifestyle modifications, such as avoiding spicy foods
Smoking	—Smoking cessation interventions
Alcohol consumption	—Moderate alcohol intake or complete abstinence
Stress	—Stress reduction techniques, lifestyle modifications (e.g., exercise, relaxation)
Hemolysis	
Inherited hemolytic disorders	—Genetic counseling, supportive care, and disease-specific treatments for underlying conditions
Autoimmune hemolytic anemia	—Immunosuppressive therapy, corticosteroids
Medication-induced hemolysis	—Discontinue or replace medications causing hemolysis —Monitor blood counts regularly
Sickle cell anemia	—Hydroxyurea, blood transfusions, pain management
Thalassemia	—Blood transfusions, iron chelation therapy
G6PD deficiency	—Avoid triggers (e.g., certain medications, foods)
Microangiopathic hemolytic anemias	—Treat underlying conditions (e.g., TTP, HUS)
Oxidative stress	—Antioxidant supplementation, lifestyle modifications
Blood transfusions	—For severe anemia, consider transfusions as needed
General considerations	
Iron deficiency anemia	—Iron supplementation, dietary changes
Routine monitoring	—Regular follow-up appointments for clinical assessments, laboratory tests, and imaging studies as needed
Patient education	—Provide information on symptoms, treatment adherence, and lifestyle modifications
Multidisciplinary care	—Collaborate with gastroenterologists, hematologists, and other specialists for comprehensive patient management

G6PD = glucose-6-phosphate dehydrogenase, *H pylori* = *Helicobacter pylori*, Hus = hemolytic uremic syndrome, NSAIDs = nonsteroidal anti-inflammatory drugs, PPIs = proton pump inhibitors, TTP = thrombotic thrombocytopenic purpura.

## 6. Diagnostic considerations

Diagnostic considerations for peptic ulcers and hemolysis involve distinct approaches due to their different anatomical sites and pathophysiological mechanisms. However, certain diagnostic considerations and tests are essential for accurate identification and differentiation between these conditions.^[30]^ Direct visualization of the upper gastrointestinal tract using an endoscope allows for the detection of peptic ulcers. Biopsies can also be taken during endoscopy for *H pylori* testing or to rule out other potential causes of ulcers. Imaging techniques like X-rays, CT scans, or MRI scans may be used to visualize the stomach and duodenum, especially if endoscopy is contraindicated or inconclusive. Various tests such as blood antibody tests, stool antigen tests, urea breath tests, and tissue biopsy tests during endoscopy are employed to detect *H pylori* infection. Blood tests to assess for anemia (CBC) or evaluate for possible causes of bleeding, such as stool tests for occult blood, may be conducted to investigate complications like bleeding ulcers. Examination of tissue samples obtained via endoscopy helps identify the underlying cause of ulcers and differentiate between various ulcer types (e.g., *H pylori* -related, NSAID-induced, or stress-related ulcers).

## 7. Diagnostic considerations for hemolysis

Blood tests assessing RBC count, hemoglobin levels, hematocrit, and RBC indices (mean corpuscular volume, mean corpuscular hemoglobin) are fundamental to assess for anemia and clues of hemolysis.^[31]^ Microscopic examination of a blood smear enables visualization of RBC morphology, identifying abnormal shapes or features suggestive of hemolytic disorders. Measurement of immature RBCs (reticulocytes) in the blood reflects the bone marrow response to increased RBC destruction and aids in assessing the regenerative capacity of the bone marrow. These tests help diagnose autoimmune hemolytic anemias by detecting antibodies attached to the surface of RBCs. Increased bilirubin (particularly unconjugated) and decreased haptoglobin levels are indicative of increased RBC breakdown and support the diagnosis of hemolysis. Depending on suspected underlying causes (genetic disorders, autoimmune conditions), additional tests such as hemoglobin electrophoresis, enzyme assays, or DNA analysis may be performed. In cases where patients present with symptoms that could potentially be associated with both peptic ulcers and hemolysis (e.g., abdominal pain, anemia, or gastrointestinal bleeding), a comprehensive diagnostic workup is crucial. This would involve a detailed medical history, physical examination, and appropriate laboratory and imaging studies tailored to evaluate each potential condition adequately. Integration of findings from these investigations aids in accurate diagnosis and guides appropriate management strategies for optimal patient care.

Prompt and accurate diagnosis aids in the early identification of underlying causes, allowing for targeted interventions and preventing the progression of both peptic ulcers and hemolysis. Timely diagnosis may improve overall patient outcomes and reduce the risk of complications associated with untreated or poorly managed conditions. Distinguishing between *H pylori* infection, NSAID use, and underlying hemolytic disorders helps tailor treatment plans for each patient. Targeted interventions based on the specific contributors to ulcers and hemolysis can optimize the effectiveness of therapeutic approaches. A comprehensive diagnostic approach ensures a thorough evaluation of the patient medical history, symptoms, and laboratory findings. Improved understanding of the patient overall health enables healthcare providers to develop individualized care plans that address both peptic ulcers and hemolysis. Genetic testing provides insights into inherited hemolytic disorders, guiding genetic counseling and family planning. A better understanding of the genetic component allows for more informed decisions regarding the management of hemolytic conditions and potential preventive measures.^[31]^

## 8. Management strategies

The management strategies for peptic ulcers and hemolysis differ due to the distinct nature of these conditions. Tailored approaches are essential based on the underlying causes and severity of each condition. Proton pump inhibitors or H2-receptor antagonists prescribed to reduce stomach acid production, promote ulcer healing, and prevent recurrence. Antibiotics administered to eradicate *H pylori* infection in cases where it is identified as the causative factor. Cytoprotective Agents are medications like sucralfate may be used to protect the ulcerated area and aid in healing. Patients are advised to avoid NSAIDs, alcohol, and smoking, which can exacerbate ulcer symptoms. Recommendations may include avoiding spicy foods, caffeine, and acidic or fatty foods that can irritate the stomach lining. Intervention such as endoscopic hemostasis or surgery may be required in severe cases of bleeding. Surgical intervention is necessary for perforation repair or obstruction relief. Periodic endoscopic examinations may be recommended to assess ulcer healing and confirm eradication of *H pylori*. Long-term use of proton pump inhibitors might be necessary in certain cases to prevent ulcer recurrence, especially in individuals at high risk.^[32,33]^

Tailoring treatment plans based on the identified causes of peptic ulcers and hemolysis ensures more effective and targeted interventions. Individualized treatment approaches enhance therapeutic outcomes and reduce the risk of recurrent episodes or complications. Adequate management of both conditions reduces the risk of complications such as gastrointestinal bleeding, severe anemia, and long-term organ damage. Proactive management strategies aim to prevent or mitigate complications, improving the overall quality of life for patients. Involvement of a multidisciplinary team facilitates collaborative care, with specialists coordinating efforts to address both peptic ulcers and hemolysis. Enhanced communication and collaboration among healthcare providers contribute to more holistic patient care and a comprehensive understanding of the complex interplay between the 2 conditions. Patient education on the importance of adherence to medications, lifestyle modifications, and follow-up appointments is crucial for successful management. Improving patient understanding and involvement in their care enhances treatment compliance, leading to better outcomes and preventing relapses. Regular monitoring allows for the assessment of treatment efficacy, identification of emerging issues, and adjustment of management strategies. Long-term monitoring is essential to address evolving clinical needs, optimize treatment plans, and ensure sustained well-being.^[32,33]^

## 9. Management strategies for hemolysis

Antibiotics or antiviral medications to manage infections contributing to hemolysis. Corticosteroids, immunosuppressive agents, or other immune-modulating therapies to suppress autoantibody-mediated hemolysis. Specific treatments based on the underlying genetic condition, which might include splenectomy, transfusions, or bone marrow transplantation in severe cases. Administration of packed RBCs in cases of severe anemia or acute hemolysis. Supplements to support RBC production and replace deficiencies in certain hemolytic conditions. Monitoring of hemoglobin, reticulocyte count, bilirubin levels, and other hematological parameters to assess response to treatment and disease progression. Continual management and follow-up care to address chronic or recurring hemolytic conditions. If identified, avoiding medications, toxins, or activities that trigger hemolytic episodes. In certain cases, vaccinations against infections, particularly for individuals without a spleen or at risk of infections, to prevent hemolysis-associated complications.^[34]^ The management of both peptic ulcers and hemolysis often involves a multidisciplinary approach. Close collaboration between gastroenterologists, hematologists, primary care physicians, and other specialists is crucial for developing individualized treatment plans, optimizing care, and addressing potential complications associated with these conditions.

Identify and address the primary cause of hemolysis, such as autoimmune disorders, inherited hemolytic anemias (e.g., sickle cell disease, thalassemia), or medication-induced hemolysis. Prescribe corticosteroids to suppress the immune response in autoimmune hemolytic anemias. In cases of severe autoimmune hemolysis, immunosuppressive drugs like rituximab may be considered. Used in conditions like sickle cell anemia to reduce the frequency of vaso-occlusive crises and hemolysis. Administer transfusions to manage severe anemia and correct abnormal blood cell counts. Supplement with folic acid to support RBC production and counteract the increased demand during hemolysis. In conditions with chronic hemolysis and iron overload (e.g., thalassemia), iron chelation therapy may be necessary to prevent complications from excess iron. Identify and avoid triggers that can induce hemolysis in individuals with glucose-6-phosphate dehydrogenase deficiency, such as certain medications and foods. Provide pain relief for individuals with conditions associated with painful hemolysis, such as sickle cell anemia. Maintain adequate hydration to reduce the risk of vaso-occlusive crises in conditions like sickle cell disease. Identify and minimize exposure to factors that can trigger hemolysis, such as certain medications, infections, or oxidative stressors. Regularly monitor CBC and reticulocyte counts to assess the severity of hemolysis and the body compensatory response. Periodic assessments may include hemoglobin levels, hematocrit, and markers of hemolysis, depending on the underlying condition. Offer genetic counseling to individuals with inherited hemolytic disorders, providing information about the condition, inheritance patterns, and family planning options. Educate patients about their specific hemolytic condition, triggers to avoid, and the importance of adherence to prescribed medications and follow-up appointments. Collaborate with hematologists and other specialists to ensure comprehensive care, especially in complex cases with overlapping conditions or comorbidities.^[34]^

## 10. Implications for clinical and health policy making

The implications of understanding the relationship between peptic ulcers and hemolysis have significant ramifications for both clinical practice and health policy formulation, offering opportunities to enhance patient care and resource allocation. Some key implications include.

### 10.1. Clinical implications

Integrated screening and evaluation: Healthcare providers should consider a comprehensive approach when evaluating patients presenting with symptoms suggestive of both peptic ulcers and hemolysis. Integration of diagnostic assessments for gastrointestinal and hematological disorders might lead to earlier identification and appropriate management of these conditions.

Multidisciplinary collaboration: Given the potential shared risk factors and complex presentations, fostering collaboration among gastroenterologists, hematologists, primary care physicians, and other specialists becomes crucial. This multidisciplinary approach can facilitate comprehensive evaluations and tailored treatment strategies for patients with overlapping conditions.

Targeted management: Understanding potential pathophysiological links could guide more targeted therapeutic interventions. For instance, in cases where both peptic ulcers and hemolysis coexist, management strategies could aim at addressing inflammatory responses effectively while managing specific complications of both conditions concurrently.

Long-term monitoring: Patients with coexisting peptic ulcers and hemolysis may require long-term monitoring for ulcer healing, *H pylori* eradication, hemoglobin levels, and other hematological parameters. Ensuring regular follow-ups helps track disease progression, treatment responses, and potential complications.

### 10.2. Health policy implications

Guideline development: Evidence-based guidelines should consider the potential associations between peptic ulcers and hemolysis, incorporating recommendations for integrated evaluations and management approaches for individuals with concurrent or overlapping symptoms.

Resource allocation: Health policies focused on resource allocation might need adjustments to accommodate the need for integrated diagnostic tests and multidisciplinary care for patients with potential overlaps between peptic ulcers and hemolysis.

Education and training: Healthcare professionals require ongoing education and training to recognize the intersections between these conditions, fostering a more comprehensive understanding and improving patient care through collaborative practices.

Research funding: Encouraging research initiatives to explore the underlying mechanisms linking peptic ulcers and hemolysis could lead to novel therapeutic targets and interventions. Funding directed toward investigating shared pathways and potential interventions may contribute to improved patient outcomes.

Understanding and acknowledging the potential connections between peptic ulcers and hemolysis can have far-reaching implications, influencing clinical decision-making, care coordination, guideline development, resource allocation, and research priorities. Implementation of these implications in clinical practice and health policy settings can ultimately lead to enhanced patient care and better outcomes for individuals affected by these conditions.

## 11. Conclusion

In conclusion, exploring the intricate relationship between peptic ulcers and hemolysis reveals a nuanced interplay between seemingly distinct medical conditions. While traditionally studied and managed separately, emerging insights suggest potential links, shared mechanisms, and clinical implications that warrant attention. The pathophysiological links, such as chronic inflammation, oxidative stress, and potential immunological influences, hint at possible associations between peptic ulcers and hemolysis. Shared risk factors, including medication use, infectious agents like *H pylori*, autoimmune phenomena, and genetic predispositions, suggest overlapping vulnerabilities in affected individuals.

Diagnostic considerations and management strategies, though distinct for each condition, underscore the importance of a comprehensive approach when patients present with overlapping symptoms or potential coexistence of peptic ulcers and hemolysis. Integrated diagnostic evaluations, multidisciplinary collaboration, and targeted treatment approaches are pivotal in optimizing patient care. From a clinical standpoint, understanding these potential intersections calls for heightened awareness among healthcare providers, emphasizing the need for thorough assessments and tailored management strategies. Multidisciplinary collaboration among gastroenterologists, hematologists, and other specialists becomes paramount in providing comprehensive care to individuals with potential overlaps between these conditions.

## Author contributions

**Conceptualization:** Emmanuel Ifeanyi Obeagu.

**Methodology:** Emmanuel Ifeanyi Obeagu, Getrude Uzoma Obeagu.

**Supervision:** Emmanuel Ifeanyi Obeagu.

**Visualization:** Emmanuel Ifeanyi Obeagu.

**Writing – original draft:** Emmanuel Ifeanyi Obeagu, Getrude Uzoma Obeagu.

**Writing – review & editing:** Emmanuel Ifeanyi Obeagu, Getrude Uzoma Obeagu.
